# Usefulness of F-18 FDG PET/CT in subcutaneous panniculitis-like T cell lymphoma: disease extent and treatment response evaluation

**DOI:** 10.2478/v10019-012-0017-z

**Published:** 2012-11-09

**Authors:** Jin-Suk Kim, Young Jin Jeong, Myung-Hee Sohn, Hwan-Jeong Jeong, Seok Tae Lim, Dong Wook Kim, Jae-Yong Kwak, Chang-Yeol Yim

**Affiliations:** 1 Department of Nuclear Medicine, Konyang University Hospital, Daejeon, South Korea; 2 Department of Nuclear Medicine, Dong-A University Medical Center, Busan, South Korea; 3 Department of Nuclear Medicine; 4 Department of Internal Medicine and; 5 Research Institute of Clinical Medicine, Chonbuk National University Medical School and Hospital, Jeonju, Jeonbuk, South Korea

**Keywords:** subcutaneous panniculitis-like T-cell lymphoma, F-18 FDG, PET/CT

## Abstract

**Background.:**

Subcutaneous panniculitis-like T-cell lymphoma (SPTCL) is a rare form of cutaneous lymphomas, accounting for less than 1% of cases of non-Hodgkin’s lymphoma. Fluorine-18 fluorodeoxyglucose (F-18 FDG) positron-emission tomography/computed tomography (PET/CT) findings of SPTCL before and after treatment were rarely reported.

**Case report.:**

We report a case of SPTCL in which F-18 FDG PET/CT showed increased FDG accumulations in numerous subcutaneous nodules without extracutaneous disease. Contrast-enhanced CT during F-18 FDG PET/CT showed multiple minimally enhancing nodules with an infiltrative pattern in the subcutaneous layer throughout the body. Follow-up F-18 FDG PET/CT after three cycles of CHOP chemotherapy showed a complete metabolic remission of the lesions.

**Conclusions.:**

F-18 FDG PET/CT is suggested to be useful in assessing the disease activity, extent and treatment response in SPTCL.

## Introduction

Subcutaneous panniculitis-like T-cell lymphoma (SPTCL) is a rare disorder that is often confused with panniculitis. Patients usually present with multiple subcutaneous nodules on the extremities and trunk without visceral disease. Systemic symptoms such as fever, fatigue, and weigh loss may be present. The disease may be complicated by hemophagocytic syndrome, which is often associated with a rapidly progressive course.^1^ The diagnosis of SPTCL is currently based on clinical and histological findings.^2^ CT is a noninvasive imaging modality that is widely used for staging in patients with lymphoma^3^, but it does not provide much information in cutaneous lesions.^4^

We report herein a patient with SPTCL in whom F-18 FDG PET/CT was valuable in assessing the extent of the disease and the treatment response.

## Case report

A 30-year-old man presented with a 10-year history of multiple subcutaneous nodules on the abdominal wall which were rubbery on palpation. The nodules were slowly enlarging in size. He was otherwise healthy without weight loss, fever, or chill. On admission, his laboratory tests showed elevated levels of serum lactate dehydrogenase and β2 –microglobulin.

**FIGURE 1:**
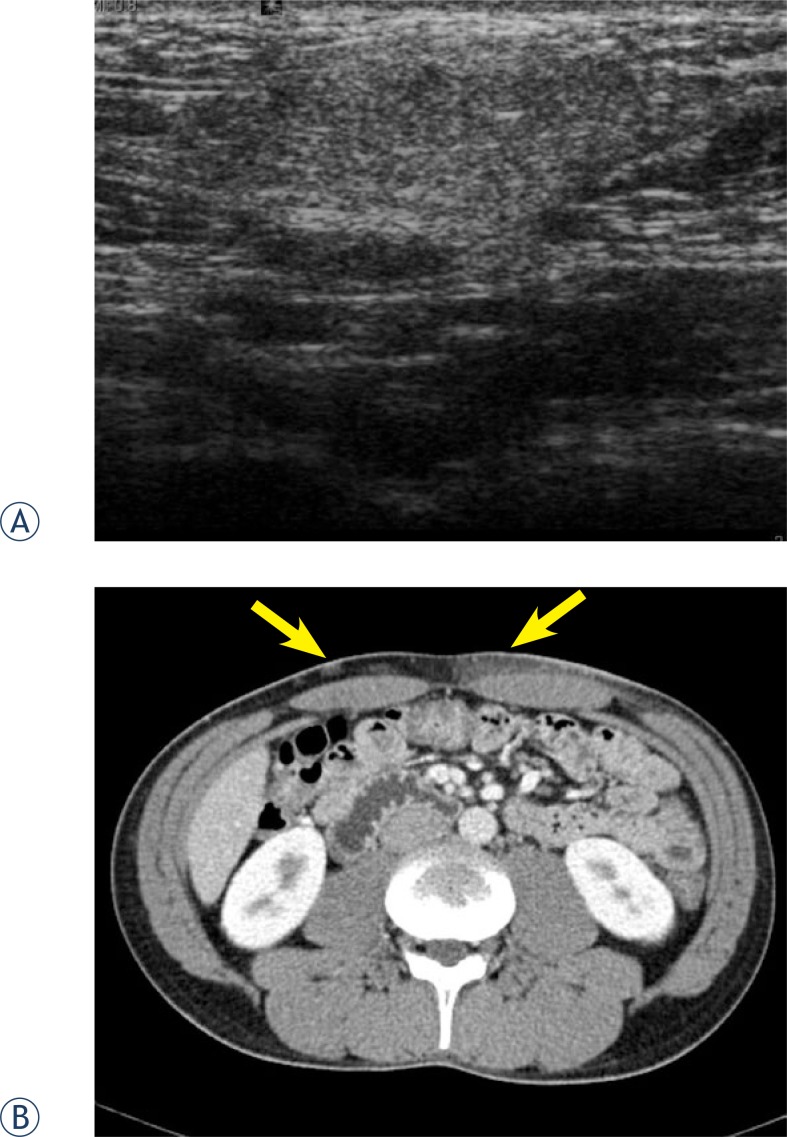
On gray-scale US examination of the lesion in the left abdomen shows ill-defined hyperechoic infiltration (A). Contrast enhanced CT shows multiple mild enhancing nodular and diffuse infiltrative lesions (arrows) in the subcutaneous layer of the anterior abdomen (B).

Ultrasonography was performed on the abdomen. The lesions revealed an ill-defined hyperechoic infiltration (Figure 1A). On abdominal and pelvic CT scanning with contrast enhancement, there were multiple slightly enhancing infiltrative nodular or non-nodular lesions in the subcutaneous layer of the abdominal wall (Figure 1B). One of subcutaneous nodules of the abdominal wall was surgically excised. On histopathologic examination of the excised tissue revealed lymphoid cells diffusely infiltrating through the subcutaneous tissue on hematoxylin & eosin staining. On immunohistochemical staining, the tumor cells were positive for CD3, CD4 and CD8, but negative for CD56. These histologic findings were consistent with SPTCL.

**FIGURE 2:**
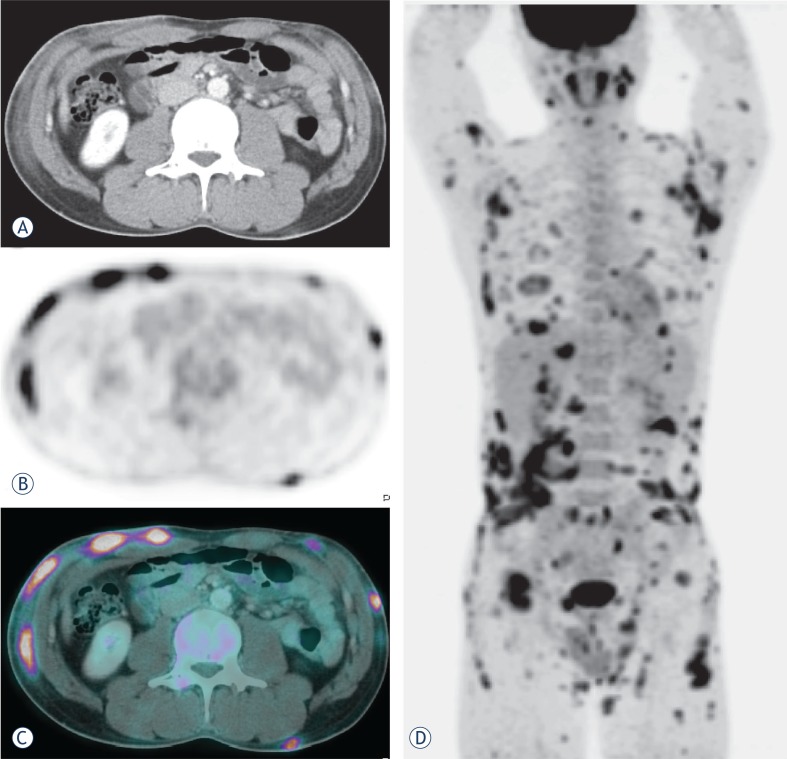
Axial contrast-enhanced CT (A), PET (B), and fusion PET/CT (C) images performed for initial staging demonstrated areas of abnormal increased F-18 FDG uptake corresponding to infiltrative changes in the subcutaneous adipose tissue of the anterior abdominal wall. Extensive disease involvement throughout the body with numerous subcutaneous nodules is best visualized on the maximum intensity projection (MIP) image (D).

For systemic surveillance, F-18 FDG PET/CT was performed. Images were obtained 1 hour after an intravenous injection of F-18 FDG (440 MBq) using a PET/CT scanner (Biograph 16 LSO Hi-Res, Siemens, Germany). The patient fasted for 6 hours: the serum glucose level measured before examination was 92 mg/dl. F-18 FDG PET/CT images revealed multiple increased F-18 FDG uptakes corresponding to the infiltrative lesions in the subcutaneous adipose tissue of the chest, back, abdomen and both extremities (Figure 2A–D). However, there was no evidence of lymph node involvement.

**FIGURE 3:**
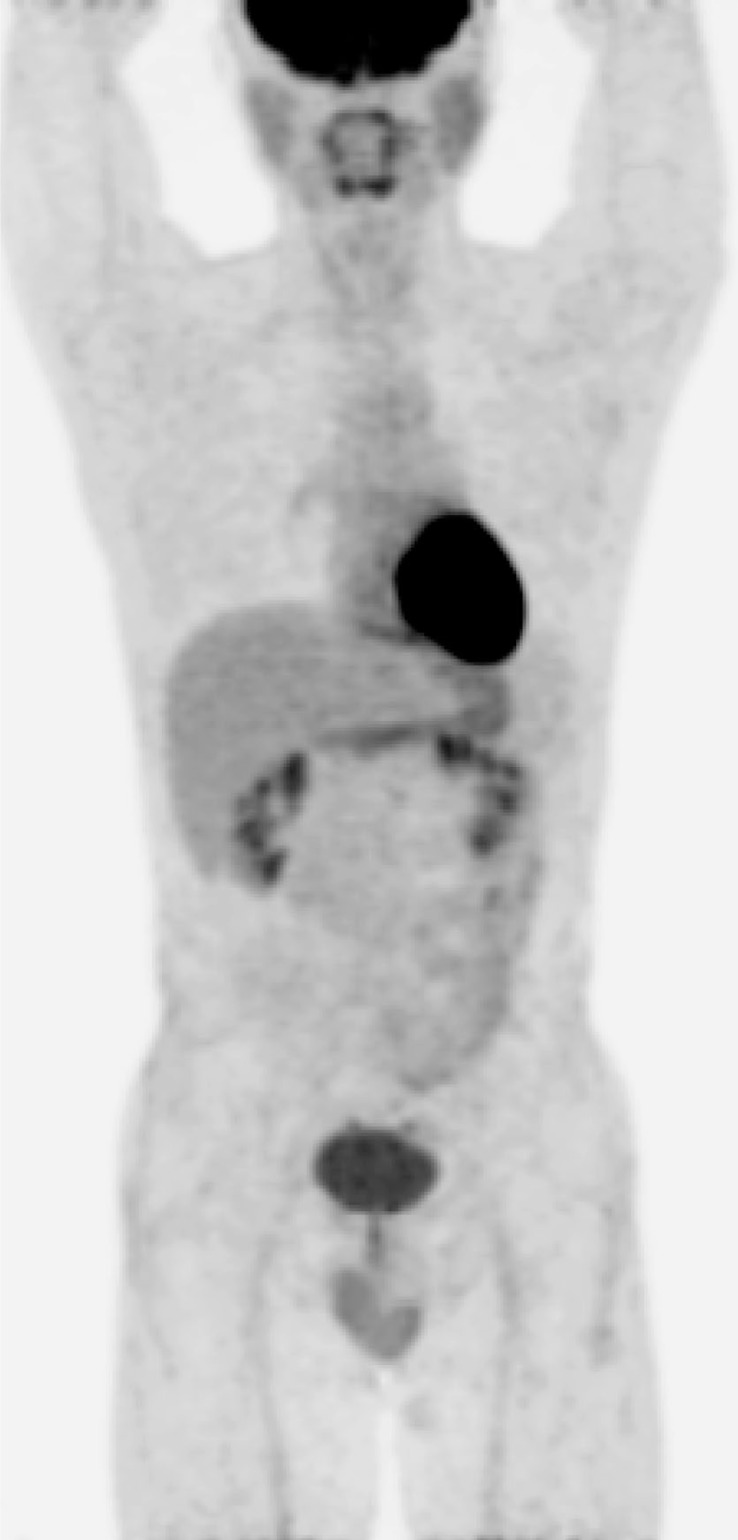
After three cycles of chemotherapy, the MIP image of follow-up F-18 FDG PET/CT showed complete metabolic remission of the involved lesions.

The patient received three cycles of CHOP (Cyclophosphamide, Adriamycin, Vincristine and Prednisolone) chemotherapy. After the chemotherapy, a follow-up F-18 FDG PET/CT scan showed a complete metabolic remission of the previous lesions (Figure 3). The patient then received additional three cycles of CHOP (total 6 cycles), and maintained the complete remission with the resolution of all skin lesions. Currently, the patient has been well without recurrence for three years after the last dose of CHOP chemotherapy.

## Discussion

According to the World Health Organization (WHO) classification, lymphoid malignancies are divided largely into T- and B-cell lymphomas, and Hodgkin’s disease. T-cell lymphomas are divided into precursor T-cell lymphomas and peripheral T-cell lymphomas. In the category of peripheral T-cell lymphomas, SPTCL has a very low incidence, accounting for less than 1% of cases of non-Hodgkin’s lymphoma.^5^ It was classified as a subtype of cutaneous T-cell lymphoma that preferentially infiltrates the subcutaneous tissue without overt lymph node involvement.^2^

SPTCL occurs in adults as well as in young children, and both sexes are equally affected. Patients present with multiple subcutaneous nodules and plaques, which progress and may ulcerate.^6^,^7^ Systemic symptoms of this disease are variable, and fever, fatigue, and weight loss may be present. Dissemination to extracutaneous sites is rare. SPTCL may be preceded for years or decades by a seemingly benign panniculitis.^8^–^10^ Two clinical courses are reported: Some patients have a protracted course of recurrent spontaneously healing subcutaneous nodules without systemic signs or symptoms; other patients have a rapidly progressive disease and very poor outcome^11^, which is usually due to the development of hemophagocytic syndrome or hepatic dysfunction.

Histologically, SPTCL is characterized by lymphocytic infiltrates confined primarily to the fat lobules in the subcutaneous tissue.^2^,^4^,^12^ Rimming of individual fat cells by neoplastic T-cells is a helpful, though not completely specific, diagnostic feature.^13^ In early stage disease, the neoplastic infiltrates may lack significant atypia and a heavy inflammatory infiltrate may predominate.^8^–^10^

On CT examination, multiple enhancing nodules are well recognized in the subcutaneous layer of the involved body site.^14^ However, these findings are also noted in inflammatory panniculitis associated with systemic lupus erythematosus or rheumatoid arthritis, subcutaneous metastases from malignant melanoma or breast cancer, and nodules originating from bacterial and fungal infections or from parasitic infestations.^15^

The F-18 FDG PET/CT has emerged as the standard of care for staging, monitoring of the response to therapy, and the detection of disease recurrence in the majority of oncological patients as well as in patients with Hodgkin’s and aggressive non-Hodgkin’s lymphomas.^16^–^18^ However, limited studies have been published in the literature on the value of F-18 FDG PET or PET/CT for the evaluation of cutaneous lymphoma. Moreover, only a few cases of SPTCL have been reported describing the findings of F-18 FDG PET or PET/CT. Most studies demonstrated that the subcutaneous lesions of SPTCL showed an FDG-avidity and indicated the superiority of PET/CT over CT alone in detecting nodal involvement.^19^–^22^ In our case of SPTCL, F-18 FDG PET/CT detected many lesions with greater sensitivity than did physical examination or CT. These studies reported that F-18 FDG PET was valuable in monitoring the treatment response and detecting the extracutaneous lesion in SPTLC.^19^,^23^

Although further evaluations are needed, the findings in our case suggest that F-18 FDG-PET/CT is a valuable tool for diagnostic work-up, staging and response monitoring in patients with SPTCL as well as those with other FDG-avid lymphoma.
